# Garlicinals A–D: Bioactive Organosulfur α,β-Unsaturated Aldehydes from Garlic (*Allium sativum* L.) Hydrolate

**DOI:** 10.1021/acsomega.5c09432

**Published:** 2025-11-12

**Authors:** Alberto Galisteo, José F. Quílez del Moral, Alejandro F. Barrero, María F Andrés, Azucena Gonzalez-Coloma

**Affiliations:** † Department of Chemistry, University of Huelva, Huelva 21007, Spain; ‡ Department of Organic Chemistry, Institute of Biotechnology, University of Granada, Granada 18071, Spain; § Institute of Agricultural Sciences, CSIC, Madrid 28006, Spain

## Abstract

Four sulfur-derived compounds, garlicinals A–D (**1–4**), were isolated from the garlic (*Allium sativum*) bulb hydrolate. The structural elucidation of **1–4** was achieved with the help of HRMS and 1D and 2D NMR. All of these compounds are characterized by an α,β-unsaturated aldehyde in their structure. Noteworthy, this structural motif is unique in garlic-derived compounds, which would convert them into a new family of garlic-derived organosulfur compounds. A synthetic path to these compounds is proposed. Garlicinals **A–C** showed potent fungicidal activity against plant pathogens (*Aspergillus niger* and *Botrytis cinerea*). Furthermore, **1–4** were active against the nematode *Meloidogyne javanica*.

## Graphical abstract

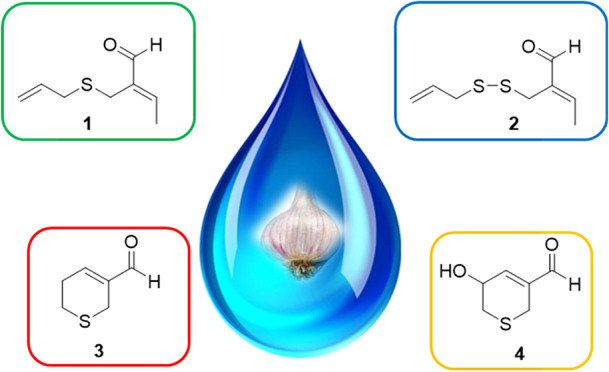

## Introduction

The widespread use of synthetic pesticides over the past few decades has contributed significantly to agricultural productivity by controlling pests, weeds, and diseases. However, the growing body of evidence linking synthetic pesticides to serious environmental damage and human health concerns, including endocrine disruption, carcinogenicity, and biodiversity loss, has led to increasing public and scientific scrutiny.^1–6^ In response, many countries are implementing or proposing stricter regulations to limit or phase out the use of conventional chemical pesticides.^7^ These regulatory trends, combined with increasing consumer demand for sustainable and eco-friendly agricultural practices, are driving a renewed interest in the development of biopesticides derived from natural products.^8^

Among the natural sources being explored, hydrolates (aromatic water residues obtained during the steam distillation of essential oils) have recently gained attention. Historically considered a waste product of essential oil extraction, hydrolates are now recognized for their complex chemical profiles and diverse biological activities, including antimicrobial, antifungal, insecticidal, and nematicidal effects.^9,10^ Their valorization represents a promising example of a circular bioeconomy, where agro-industrial residues are transformed into value-added products.

Considering that global garlic production exceeded 28 million metric tons in 2023,^11^ the potential for industrial-scale generation and utilization of garlic hydrolate is significant. In this context, we previously demonstrated the nematicidal potential of the organic extract of garlic (*Allium sativum*) hydrolate, a byproduct of essential oil production, against the root-knot nematode *Meloidogyne javanica*.^12^

Based on these findings, this study aims to deepen our understanding of the bioactive properties of garlic hydrolate, focusing on its chemical composition and its nematicidal activity against *M. javanica*. Additionally, we explore its antifungal activity against the phytopathogenic fungi *Botrytis cinerea* and the pathogen *Aspergillus niger*. Particular attention is given to the isolation and structural identification of the active compounds responsible for the observed effects, with the ultimate goal of supporting the development of novel, environmentally friendly biopesticides derived from agro-industrial residues.

## Results and Discussion

### Chemical Study of the Hydrolate Extract

The hydrolate residue from the steam distillation of garlic bulbs was extracted as described in the Experimental Part to give an ethyl acetate (EtOAc) extract.^12^ Fractionation of the hydrolate extract on silica gel 60 gave a fraction of known compounds (fraction **A**), a second fraction (fraction **B**) containing mainly compounds **1 + 2** (4:1 ratio), and three additional fractions, fraction **C** containing mainly the oxygenated organosulfur compound **3** and fraction **E** containing compound **4**. Semipreparative HPLC separation from fraction **B** allowed the isolation of **1** and **2** in a pure form, whereas compounds **3** and **4** were isolated from fractions **C** and **E**, respectively. Compounds **1–3** are described for the first time.

The first fraction, **A**, was analyzed by GC-MS (Table 1). Diallyl disulfide (DADS, 28.6%), methyl allyl trisulfide (MATS, 21.3%), and diallyl trisulfide (DATS, 30.60%) represented almost an 80% of the total composition of this fraction. These compounds are among the main components of the essential oil and their activity is widely known.^13^

**Table 1:** Chemical Composition of Fraction **A** as Determined by GC-MS Analysis

retention time (min)	area (%)	compound
2.99	1.20	diallyl sulfide
3.58	3.87	methyl allyl disulfide
4.33	1.26	dimethyl trisulfide
6.11	28.59	diallyl disulfide (DADS)
6.42	1.61	diallyl disulfide isomer A
6.53	3.76	diallyl disulfide isomer B
7.31	21.27	methyl allyl trisulfide (MATS)
8.64	1.74	4H-1,2,3-trithiine
8.91	2.53	2-vinyl-4H-1,3-dithiine
10.84	30.60	diallyl trisulfide (DATS)

Compound **1** (Figure 1) showed, in its HRMS, the molecular ion [M + H]^+^ at *m*/*z* 157.0687, corresponding to a molecular formula of C_8_H_13_OS. Analysis of its ^1^H NMR spectrum indicates the presence of an S–CH_2_–CH=CH_2_ moiety, as deduced from the values of the δ, as well as coupling constants of the signals appearing at a δ 5.86, 5.22, 5.15, and 3.18 ppm (Table 2), and the data were comparable to those exhibited by known compounds, such as diallyl sulfide (DAS).^14^ Other ^1^H and ^13^C NMR signals at δ 9.43 (s), 6.72 (q, *J* = 7.1 Hz), 193.42 ppm (CH), and 151.44 (CH), respectively, confirmed the presence of an α,β-unsaturated aldehyde with a trisubstituted double bond. The multiplicity of the olefinic proton (q, *J* = 7.1 Hz) indicated the presence of a vicinal methyl, the latter appearing at 2.09 ppm (d, *J* = 7.1 Hz).^15^ Finally, the singlet signal at δ 3.36 ppm could be attributed to one methylene group bound to sulfide. Assignment of the structure of 2-((allylthio)­methyl)-but-2-enal to **1** was confirmed on the basis of the long-range correlations observed in the HMBC experiment (Table 2, Figure 1).

**Figure 1:**
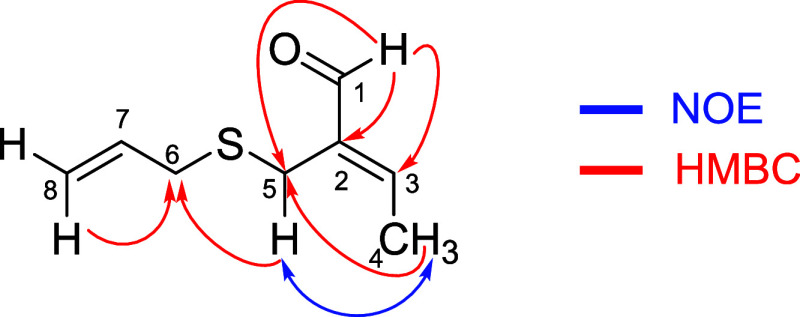
Key HMBC and NOE correlations for garlicinal A (**1**).

**Table 2:** NMR Data of Compound **1**

position	^1^H (ppm)	^13^C (ppm)^a^	NOE	HMBC
1	9.43 s	193.42 (CH)	H_3_	C_2_, C_3_, C_5_
2		141.60 (C)		
3	6.72 q (*J* = 7.1 Hz)	151.44 (CH)	H_1_, H_4_	C_1_, C_4_, C_5_
4	2.09 d (*J* = 7.1 Hz)	15.25 (CH_3_)	H_3_, H_5_	C_1_, C_2_, C_3_, C_5_
5	3.36 s	22.98 (CH_2_)	H_4_, H_6_	C_1_, C_2_, C_3_, C_6_
6	3.18 dt (*J* = 7.0, 1.2 Hz)	35.69 (CH_2_)		C_5_, C_7_, C_8_
7	5.86 ddt (*J* = 17.0, 10.0, 7.0 Hz)	134.18 (CH)		C_6_
8_α_	5.22 dq (*J* = 17.0, 1.2 Hz)	117.35 (CH_2_)		C_6_, C_7_
8_β_	5.15 dq (*J* = 10.0, 1.2 Hz)	117.35 (CH_2_)		C_6_

a HSQC-based assignment.

The geometry of the trisubstituted double bond was established after the analysis of the 1D NOE experiments. Thus, the observation of NOE at H4 (methyl group) after irradiation of H5 confirmed the *E* geometry of this unsaturation. This geometry is corroborated after noticing the γ-shielding effect experienced by C5.^15^

Then, compound **1** was completely identified as 2-((allylthio)­methyl)­but-2*E*-enal and called garlicinal A.

Compound **2** (Figure 2) was assigned to the molecular formula C_8_H_13_OS_2_ from its HRMS ([M + H]^+^, *m*/*z* 189.0399). Its NMR spectra were very similar to those of **1**, with the main differences being the chemical shifts of the carbons bound to the sulfur atom. Thus, while these carbons resonate in compound **1** at δ 35.69 and 22.98 ppm (Table 2), the corresponding resonances appeared in compound **2** at δ 41.91 and 30.78 ppm, which together with the molecular formula suggested the presence of a disulfide bridge in compound **2.** The relative stereochemistry was determined as *E* based on the NOEs found at H5 and H3 after irradiating H4 and H1, respectively. This assignment was further supported after noticing that similar chemical shifts differences were found when comparing the NMR data of DAS with those of DADS or DATS.^14,16^ Compound **2** was named garlicinal B.

**Figure 2:**
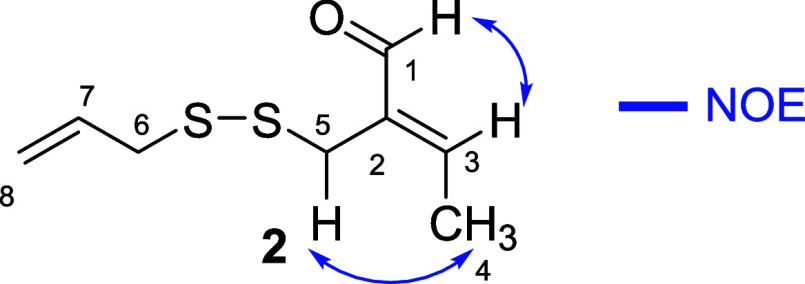
Key NOE correlations for garlicinal B (**2**).

A proposal for the formation of garlicinals **1** and **2** in a retrosynthetic way is presented in Scheme 1. Thus, their generation could be rationalized considering an aldol condensation between synthon **I** or **II** and a molecule of acetaldehyde (**III**). Synthons **I** and **II** would be, in turn, originated via Michael addition of allyl mercaptan (**IV**) or 2-propeneperthiol (**V**) to acrolein (**VI**), respectively. The fact that compounds **III** and **VI** are known to be found in garlic extracts supported this proposal.^17–19^

**Scheme 1:**
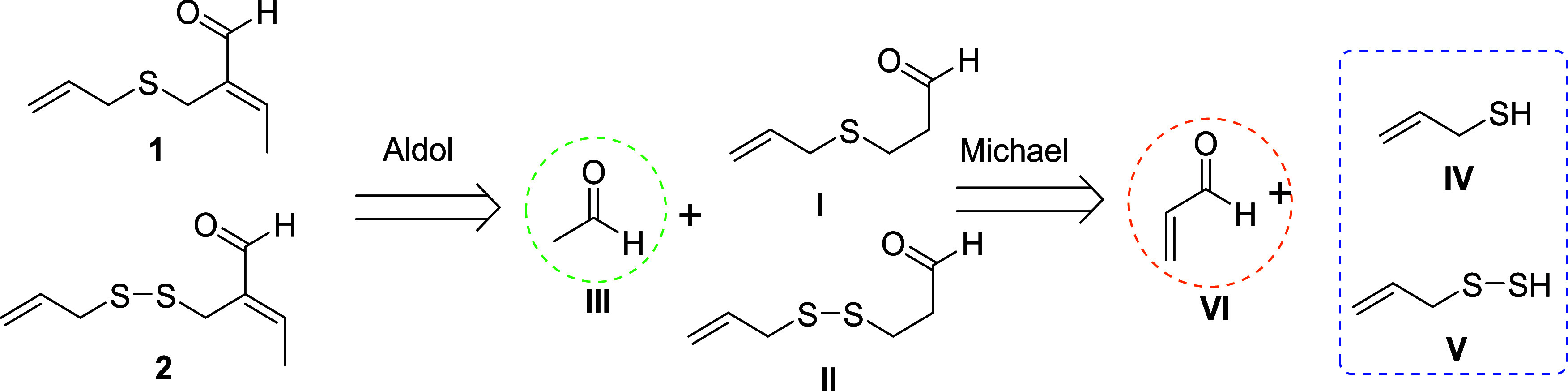
Retrosynthetic Scheme Proposed for the Generation of Garlicinals A and B (**1** and **2**)

The origin of synthons **IV** and **V** could be attributed to the aqueous degradation of allicin (**VII**) or to the reduction of polysulfides present in garlic.^20,21^ In the case of acrolein (**VI**), this compound may be originated as a result of a thermic degradation of allicin in aqueous medium (Scheme 2, (**a**)), or by the hydrolysis of cation **VIII** (Scheme 2, (**b**)), an intermediate in the synthesis of ajoene.^22^ A third way could be the thermic or photochemical degradation of cysteine.^23,24^

**Scheme 2:**
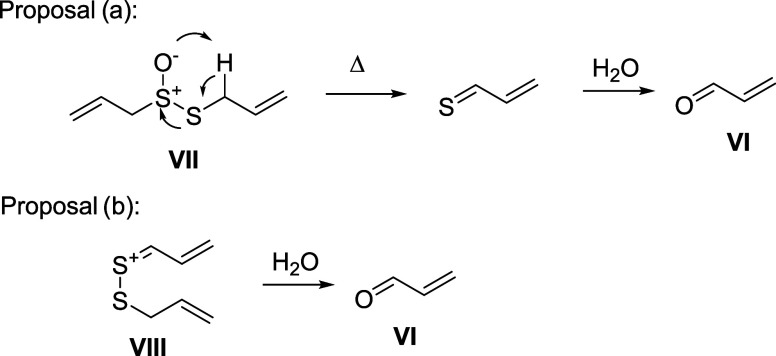
Proposals of the Generation of Acrolein (**VI**)

Starting from these synthons, the process of the formation of garlicinals **1** and **2** can take place in aqueous medium easily, where the approach of the organic synthons is favored.^25^ Under these circumstances, a multicomponent reaction (Michael–Aldol) process as shown in Figure 3 is possible.

**Figure 3:**
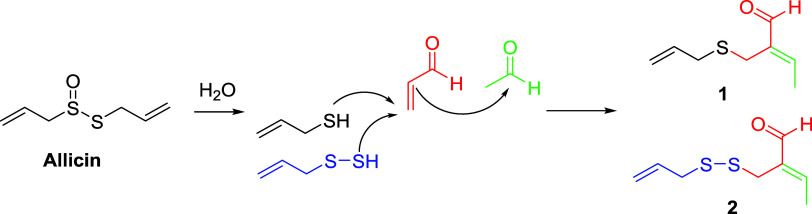
Process for the generation of garlicinals A and B (**1** and **2**).

HRMS of **3** showed a [M + H]^+^ at *m*/*z* 129.0374, which together with its ^1^H and ^13^C NMR data (Table 3), established the molecular formula C_6_H_9_OS. This molecular formula, together with the analysis of its ^13^C NMR spectrum, permitted assigning a cyclic structure for compound **3**.

**Table 3:** NMR Data of Compound **3**

position	^1^H (ppm)	^13^C (ppm)^a^	COSY	HMBC
1				
2	3.33 q (*J* = 2.2 Hz)	22.35 (CH_2_)	H_4,_ H_5_	C_3_, C_4_
3		138.99 (C)		
4	6.91 m	150.93 (CH)	H_2,_ H_5_	C_6_, C_7_
5	2.72–2.65 m	27.53 (CH_2_)	H_4_, H_6_	C_3_, C_4_, C_6_
6	2.80 t (*J* = 5.7 Hz)	24.72 (CH_2_)	H_5_	C_2_, C_4_, C_5_
7	9.38 s	192.78 (CH)		C_2_, C_3_

a HSQC-based assignment.

The ^1^H NMR signals at δ 9.38 and 6.91 ppm suggested the existence of an α,β-unsaturated aldehyde with a trisubstituted double bond in the structure of **3**. Signals corresponding to the three methylene groups complete the ^1^H NMR spectrum. Two of these methylene groups were assigned to be bound to the sulfur atom (H_6_, 2.80 t (*J* = 5.7 Hz)); H_2_, 3.33 q (*J* = 2.2 Hz), whereas the remaining one was located α to the olefinic proton as confirmed after the analysis of the correlations observed in its COSY and HMBC spectra (Table 3). Compound **3** was thus assigned to the structure of 5,6-dihydro-2H-thiopyran-3-carbaldehyde named garlicinal C (Figure 4).

**Figure 4:**
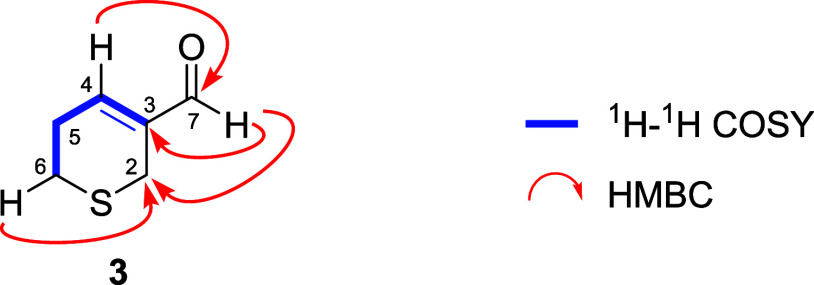
Structure of garlicinal C (**3**).

The synthesis of compound **3** was described in a number of patents,^26^ although this is the first isolation of this compound originated from a natural source, the bulbs of *Allium sativum*. The spectroscopic data of compound **3** match those reported in the literature.

The NMR spectra of compound **4** were very similar to those of **3**, with the only difference being the presence of an oxygenated methine (^1^H NMR, δ 4.59, m; ^13^C NMR, δ 65.16) instead of a methylene group. The HRMS spectrum of **4** was compared with that of garlicinal C (**3**) to corroborate the presence of an additional oxygen atom. The correlations observed in the COSY spectrum of **4**, in particular, the correlation of this oxygenated methine with the olefinic proton, allowed the oxygenated function to locate easily at C5, and consequently, compound **4** named garlicinal D was deduced as the structure of 5-hydroxy-5,6-dihydro-2H-thiopyran-3-carbaldehyde. Since the optical rotation [α]_D_ value of **4** was found to be zero, compound **4** should exist as a racemic mixture (Figure 5).

**Figure 5:**
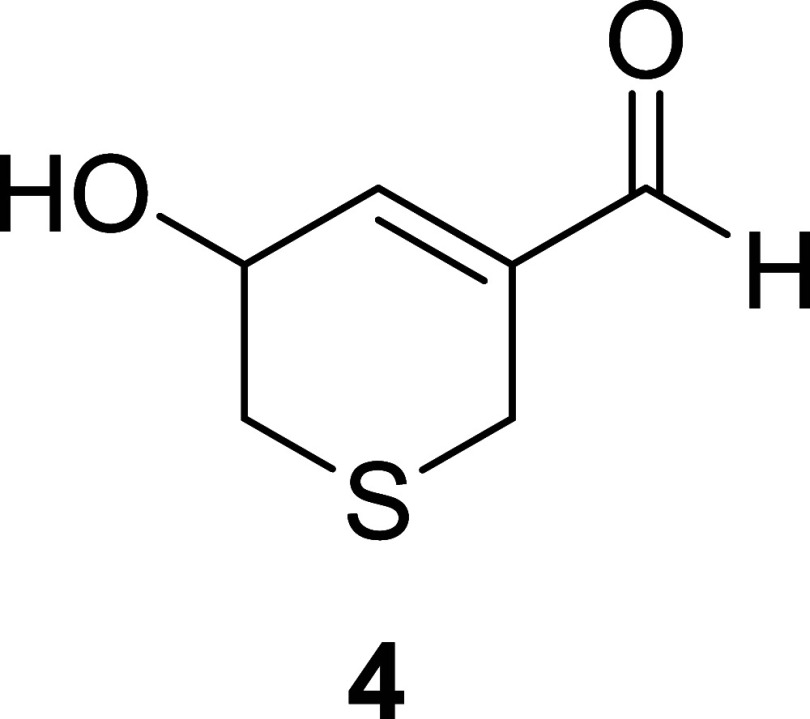
Structure of garlicinal D (**4**).

The formation of garlicinals C and D (**3** and **4**) has been rationalized as shown in Figure 6. Thus, **3** would be generated after an intramolecular aldolic reaction of the intermediate **IX**, which, in turn, would be the result of two consecutive Michael additions between two molecules of acrolein (**VI**) and one molecule of hydrogen sulfide; the latter is formed from the degradation of cysteine.^23^ From garlicinal C (**3**), an autoxidation process in several steps for the formation of the allylic alcohol is possible, thus generating garlicinal D (**4**).^27^

**Figure 6:**
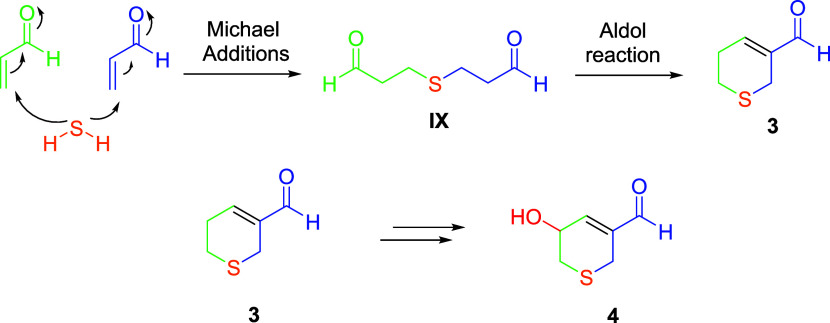
Process for the generation of garlicinals C and D (**3** and **4**).

The garlicinals **1–4** are small molecules (<10 carbons) that contain an aldehyde group. This aldehyde can be hydrated to generate a gem-diol group, that should be soluble in hot water and retain the compounds in the hydrolate. In this sense, a sustainable synthesis “on water” of these compounds could be envisaged.

The relevance of garlic as a crop together with the wide array of bioactivities associated with its metabolites can be assessed by considering the number of publications in journals, patents, reviews, and clinical trials found in SciFinder using “*Allium sativum*” as a keyword in the last 10 years. The resulting figures are 10K, 22K, 1018 and 99, respectively. Considering the amount of research on this species that these figures support, it is quite significant that, among the 70 organosulfur compounds found in garlic, this is the first report of garlic organosulfur derivatives showing an α,β-unsaturated aldehyde substructure. Bearing in mind the bioactivity inherent to this structural motif, this new family of compounds are, in our opinion, promising candidates for the discovery of new and more potent bioactive compounds.

### Bioactivity

Table 4 shows the spore germination inhibition effects of the extract, fraction **A** (diallyl disulfide DADS, diallyl trisulfide DATS, and methyl allyl trisulfide MATS mixture), fraction **B** (mainly garlicinals **1**+**2**, 4:1), and isolated garlicinals **1–4** against the phytopathogens *Botrytis cinerea* and *Aspergillus niger*.

**Table 4:** Antifungal Effects of Garlic Hydrolate Fractions **A** and **B**, and Garlicinals **1–4** against *Botrytis cinerea* and *Aspergillus niger* Spore Germination

	^a^effective dose ED_50_ (μg/mL)	
compound	*B. cinerea*	*A. niger*
extract	27.52 (18.54–40.85)	106.20 (75.83–148.72)
fraction **A**	47.78 (23.51–64.57)	86.04 (64.61–114.94)
fraction **B**	3.33 (1.64–6.74)	38.43 (24.08–61.33)
**1**	27.13 (21.09–27.12)	78.07 (63.96–95.28)
**2**	10.10 (7.59–13.44)	30.76 (24.39–38.78)
**3**	8.79 (5.36–14.43)	16.96 (7.98–36.03)
**4**	>100	>100
thymol	19.54 (22.94–15.74)	50.00 (45.30–55.20)

a Effective dose to give 50% germination inhibition.

*B. cinerea* spore germination was strongly affected by fraction B (rich in garlicinal B (**2**), ED_50_ of 3.33 μg/mL), followed by garlicinal C (**3**) (ED_50_ of 8.79 μg/mL), garlicinal B (**2**) (ED_50_ of 10.10 μg/mL), garlicinal A (**1**) (ED_50_ of 27.13) and fraction **A** (rich in DADS-DATS-MATS, ED_50_ of 47.78 μg/mL). Fraction B, a mixture of **1**+**2** (4:1), was more active (ED_50_ of 3.3 μg/mL) than its component garlicinals A (**1**, ED_50_ of 27.13) and B (**2**, ED_50_ of 10.1) tested separately. This could be attributed to a synergistic effect (Table 5). It should be noted that the presence of a second sulfur atom in garlicinal B (**2**) or cyclization in garlicinal C (**3**) increased its activity against *B. cinerea* by up to three times with respect to that of garlicinal A (**1**).

**Table 5:** Nematicidal Effects of the Garlic Hydrolate *Meloidogyne javanica*

	effective doses (μg/mL)	
fraction/compound	MLD^a^	LD_50_ ^b^
extract	125.0	110.0 (100.0–111.0)^c^
fraction **A**	31.24	12.0 (11.6–12.4)
fraction **B**	120.0	42.4 (40.3–44.6)
**3**	250.0	27.8 (8.9–41.7)
**4**	1000.0	621 (602–639)
thymol^c^	250.0	137 (131–143)

a Minimum lethal dose (MLD) to give 100% mortality.

b Effective Lethal dose to give 50% mortality.

c From Galisteo et al.^12^

Overall, *A. niger* was more resistant than *B. cinerea*, being affected by garlicinal C (**3**) (ED_50_ of 16.96 μg/mL), followed by fraction B (ED_50_ of 38.43 μg/mL), garlicinal B (**2**) (ED_50_ of 30.76 μg/mL), fraction A (ED_50_ 78.07 μg/mL), and garlicinal A (**1**) (ED_50_ of 71.87 μg/mL) (Table 5). In this case, cyclization in garlicinal C (**3**) increase its activity up to four times against *A. niger* with respect to that of garlicinal A (**1**).

Volatile sulfur compounds derived from garlic, including DADS and DATS, as well as garlic extracts have been reported as being antifungal.^28^ Garlic extracts and oils have shown activity against various crop damaging fungi that cause a huge loss in yield, including *B. cinerea* ^29,30^ and *A. niger*.^31^ However, this is the first report on the antifungal effects of the oxygenated sulfur compounds garlicinals A**–**D (**1–4**).

Table 5 shows the nematicidal activity of the EtOAc extract, fraction A (diallyl disulfide DADS, diallyl trisulfide DATS, and methyl allyl trisulfide MATS mixture), fraction **B** (compounds **1**+**2**, 4:1), and compounds **3** and **4**.

The activity of the extract showed a similar minimum lethal dose to fraction B (125 and 120 μg/mL, respectively) but was less effective based on their LD_50_ values (110 versus 42 μg/mL). The most effective nematicidal was fraction **A**, rich in DADS-DATS-MATS (MLD of 31.0 and LD_50_ of 12.0 μg/mL), followed by fraction **B** (garlicinals A-B (**1 + 2)**, 4:1) mostly composed of garlicinal B (**2**) (MLD of 120 and LD_50_ of 42.4 μg/mL), garlicinal C (**3**) (MLD of 250 and LD_50_ of 28 μg/mL), and garlicinal D (**4**) (MLD of 1000 and LD_50_ of 621 μg/mL). Fractions A and B were more active than the positive control thymol (11.4 and 3 times more based on their LD_50_ values), garlicinal C (**3**) with an MLD similar to that of thymol (250 μg/mL) was more active based on its LD_50_ value (5 times more active), and garlicinal D (**4**) was less effective. Therefore, fractions **A** and **B** explained most of the nematicidal activity of the extract.

The nematicidal activity of aqueous garlic extract has been proved against *M. incognita* DADS and DATS,^32^ the major components of garlic essential oil, garlic hydrolate extract^12^ and fraction **A**, have shown activity against the pine wood nematode, *Bursaphelenchus xylophilus* ^33^ and the root-knot nematode species, *M. javanica*,^34^ and *M. incognita*.^35^ Furthermore, the organic extract of the purple garlic essential oil-derived hydrolate, also rich in DADS and DATS, was effective against *M. javanica in vitro* and *in vivo*.^12^ However, this is the first report on the nematicidal activity of garlicinals A**–**D (**1–4)**, with the linear ones **1** and **2** being more active probably due to their higher volatility and therefore being more fumigant.

## Experimental Part

### General Methods

Silica gel 60 (35–70 μm) was used for flash column chromatography and monitored by thin-layer chromatography (TLC) carried out on 0.25 mm E. Merck silica gel plates (60F-254) using UV light as the visualizing agent and solutions of phosphomolybdic acid in ethanol. HPLC with UV and RID detection was used. Semipreparative HPLC separation was carried out on a column (5 μm silica, 9.4 mm × 250 mm) at a flow rate of 2.0 mL/min in an Agilent Series 1100 instrument. NMR spectra were performed with a Varian Direct Drive 600 (^1^H 600 MHz/^13^C 150 MHz), Varian Direct Drive 500 (^1^H 500 MHz/^13^C 125 MHz), Varian Direct Drive 400 (^1^H 400 MHz/^13^C 100 MHz), and BRUKER Avance NEO (^1^H 400 MHz/^13^C 100 MHz) spectrometers. High-resolution MS was determined on an Autospec-Q VG-Analytical (FISONS) mass spectrometer. DEPT-135 and two-dimensional (COSY, HSQC, HMBC, NOESY) NMR spectroscopy was used where appropriate to assist the assignment of signals in the ^1^H and ^13^C NMR spectra.

The volatile compounds of hydrolate fractions were analyzed by gas chromatography coupled to mass spectrometry (GC-MS) using GC-2010 (Shimadzu, Kioto, Japan) equipment coupled to a GC-MS-QP2010 (Shimadzu, Kioto, Japan) mass detector, equipped with a Simple Quadrupole analyzer, an automatic injector (AOC-20i) (Shimadzu, Kioto, Japan), and a (95%) dimethyl- (5%) diphenyl polysiloxane capillary column (30 μm × 0.25 mm ID and 0.25 μm phase thickness) (Teknokroma TRB-5, Barcelona, Spain). The samples (in DCM) were detected by electronic impact at 70 e with helium as a carrier gas. The working conditions were as follows: split mode injection (1 μL injected), division ratio (20:1), injector temperature 300 °C, transfer line temperature 250 °C, and ionization source temperature 220 °C. The initial temperature was 70 °C, heating up to 290 °C at 6 °C/min plus 20 min leaving at 290 °C. Mass spectra and retention time are used to identify compounds by comparison with the Wiley and NIST17 databases (Wiley 275 Mass Spectra Database, 2001, Palmer, Massachusetts; NIST Mass Spectra Database, 2017, Gaithersburg, Maryland).

### Plant Materials

The purple garlic plant is cultivated by Coopaman SA in Las Pedroñeras, Cuenca, Spain. At harvest, the undersize garlic bulbs (<36 mm) are considered garlic waste and were used for the preparation of the extracts.

### Extraction and Fractionation

The extract of garlic hydrolate was prepared as described previously by some of us.^12^ Eight grams of extract was column chromatographed using a mixture of solvents (hexane (H), methyl *tert*-butyl ether (MTBE) and ethyl acetate (EtOAc)) of increasing polarity to obtain five main fractions (**A**–**E**). Fraction **A** (5.5 g) was analyzed by GC-MS showing a high content of organosulfur compounds, with DADS, MATS, and DATS being the main components (GC-MS available in the Supporting Information). Fraction **B** (308 mg) was subjected to semipreparative HPLC (20 mg) to obtain garlicinal A (**1**) (9 mg, Rt = 7.84 min) and 3 mg of garlicinal B (**2**) (3 mg, Rt = 8.59 min) using H/MTBE (9–1) as the eluent. Fraction **C** (284 mg) was purified by semipreparative HPLC (20 mg) with an elution H/MTBE (8–2) to obtain garlicinal C (**3**) (16 mg, Rt = 10.31 min). Fraction **D** (176 mg) was consisted of a mixture of alcohols and fatty acids. Fraction **E** (139 mg) was chromatographed with H/MTBE (1:1) as the eluent to obtain garlicinal D (**4**) (129 mg).

Compound **1** (Garlicinal A): ^1^H NMR (600 MHz, CDCl_3_) δ 9.43 (s, 1H), 6.72 (q, *J* = 7.1 Hz, 1H), 5.86 (ddt, *J* = 17.0, 10.0, 7.0 Hz, 1H), 5.22 (dq, *J* = 17.0, 1.2 Hz, 1H), 5.15 (dq, *J* = 10.0, 1.2 Hz, 1H), 3.36 (s, 2H), 3.18 (dt, *J* = 7.0, 1.2 Hz, 2H), 2.09 (d, *J* = 7.1 Hz, 3H). ^13^C NMR (151 MHz, CDCl_3_) δ: 193.42, 151.44, 141.60, 134.18, 117.35, 35.69, 22.98, 15.25. HRMS TOF (ESI+) *m*/*z* calculated for C_8_H_13_OS [M + H]^+^ 157.0668, found 157.0687.

Compound **2** (Garlicinal B): ^1^H NMR (600 MHz, CDCl_3_) δ 9.43 (s, 1H), 6.81 (q, *J* = 7.1 Hz, 1H), 5.88 (ddt, *J* = 17.0, 10.0, 7.4 Hz, 1H), 5.23 (dq, *J* = 17.0, 1.2 Hz, 1H), 5.19 (ddd, *J* = 10.0, 1.2, 0.9 Hz, 1H), 3.64 (s, 2H), 3.36 (dt, *J* = 7.4, 0.9 Hz, 2H), 2.14 (d, *J* = 7.1 Hz, 3H). ^13^C NMR (151 MHz, CDCl_3_) δ: 192.91, 152.15, 140.35, 133.17, 118.81, 41.91, 30.78, 15.62. HRMS TOF (ESI+) *m*/*z* calculated for C_8_H_13_OS_2_ [M + H]^+^ 189.0399, found 189.0408.

Compound **3** (Garlicinal C): ^1^H NMR (600 MHz, CDCl_3_) δ 9.38 (s, 1H), 6.91 (tt, 1H), 3.33 (q, *J* = 2.2 Hz, 2H), 2.80 (t, *J* = 5.7 Hz, 2H), 2.72–2.65 (m, 2H). ^13^C NMR (126 MHz, CDCl_3_) δ: 192.78, 150.93, 138.99, 27.53, 24.72, 22.35. HRMS TOF (ESI+) *m*/*z* calcd para C_6_H_9_OS [M + H]^+^ 129.0359, found 129.0374.

Compound **4** (Garlicinal D): ^1^H NMR (400 MHz, Acetone) δ 9.44 (s, 1H), 6.85 (dq, *J* = 2.6, 1.3 Hz, 1H), 4.59 (ddtd, *J* = 8.6, 5.3, 2.7, 2.0 Hz, 1H), 3.28 (dt, *J* = 17.6, 2.4 Hz, 1H), 3.06 (dq, *J* = 17.7, 1.7 Hz, 1H), 2.90 (ddt, *J* = 13.1, 5.3, 1.3 Hz, 1H), 2.65 (dd, *J* = 13.1, 8.6 Hz, 1H). ^13^C NMR (101 MHz, Acetone) δ: 192.82, 152.67, 139.19, 65.16, 31.46, 21.36. [α]_D_ 0 (*c* = 1, DCM). HRMS TOF (ESI+) *m*/*z* calcd para C_6_H_9_O_2_S [M + H]^+^ 145.0349, found 145.0365.

### Antifungal Activity

The fungal species *Aspergillus niger* and *Botrytis cinerea* came from the fungal collection of the Instituto de Ciencias Agrarias-CSIC, Madrid, Spain, where they are maintained. The antifungal activity of the hydrolate extract and fractions were determined using a modified spore germination inhibition growth assay.^36^ The hydrolate extract or fractions were dissolved in dimethyl sulfoxide (DMSO) at 1% and evaluated at the final concentrations indicated. The spore suspensions were 7.5 × 10^5^ cells/mL in NaCl 0.9% for *A. niger* and 1 × 10^7^ cells/mL in distilled water for *B. cinerea*. Thymol (5 μg/mL) was used as a positive control at the same concentration. The samples and spore suspensions (4 replicates) were placed on 96-well plates and incubated for 24 h (28 °C for *A. niger* and 25 °C for *B. cinerea*). After the incubation process, 25 uL of an MTT (5 mg/mL) plus menadione (1 mM) solution in RMPIMOPS were added, the plates were incubated again for 3 h, the medium was removed, 200 μL of acidic isopropanol (95% isopropanol and 5% 1 M HCl) was added, and the plates were incubated for another 30 min. The absorbance was read at 490 nm in an Elisa reader. The IC_50_ values (the effective dose to give 50% inhibition) were calculated by a regression curve of % spore germination inhibition on a log dose.

### Nematicidal Activity

Hand-picked egg masses of *M. javanica* from tomato roots incubated in a water suspension at 25 °C for 24 h were used to obtain second-stage juveniles (J2). Bioassays were carried out in 96-well plates (BD Falcon, San Jose, CA), and treatments were replicated four times as described by Andrés et al.^37,38^

The fractions of the hydrolate or the compound isolates were dissolved in a 5% DMSO-Tween solution in water (0.5% Tween 20 in DMSO) and added (5 μL) to 95 μL of water containing 90–150 nematodes to give a final concentration of 1 mg/mL.^39^ The control wells contained water/DMSO/Tween 20. The positive control was thymol (LC_50_ = 0.143 mg/mL).

The plates were covered and maintained in the dark at 25 °C and the dead J2 counted at 24, 48, and 72 h under a binocular microscope. The nematicidal activity results are presented as percent dead J2s corrected according to the Schneider–Orelli’s formula.^39^ Six serial concentrations (1–0.031 mg/mL) of each treatment were tested to obtain an effective lethal concentration (LC_50_ and LC_90_) by Probit analysis (STATGRAPHICS Centurion XVI, version 16.1.02, The Plains, Virginia).

## Conclusions

Four new organosulfur compounds, namely, garlicinals A–D (**1–4**), were isolated from the industrial agrowaste hydrolate of garlic. All of these compounds share the presence of an α,β-unsaturated aldehyde in their structure, which led us to classify these compounds as a new family of oxygenated organosulfur compounds derived from garlic. A synthetic route to the generation of these compounds on water is hypothesized, which supports the feasibility of implementing a sustainable system for these compounds. Finally, these garlicinals (**1–4**) presented potent nematicidal and fungicidal activity, which support the crop protection potential of garlic hydrolate as nematicidal and antifungal. Bearing in mind the bioactivity inherent to this structural motif, this new family of compounds are, in our opinion, promising candidates for the discovery of new and more potent bioactive compounds.

## Supplementary Material



## Notes

NMRs spectrum and GC-MS chromatograms (PDF)
