# Supplementary material for: Garlicinals A–D: Bioactive Organosulfur α,β-Unsaturated Aldehydes from Garlic (Allium sativum L.) Hydrolate
Source: ACS Omega. 2025 Nov 12;10(46):56708–14. doi: 10.1021/acsomega.5c09432 (PMC12658810; doi:10.1021/acsomega.5c09432)
Supplement: Supplementary file 1 [file ao5c09432_si_001.pdf]

Supporting information.

Garlicinals A-D: Bioactive Organosulfur α,β-Unsaturated Aldehydes from Garlic (*Allium sativu*m L.) Hydrolate.

- NMR Spectrum: 1-12
- GC-MS Spectrum: 13

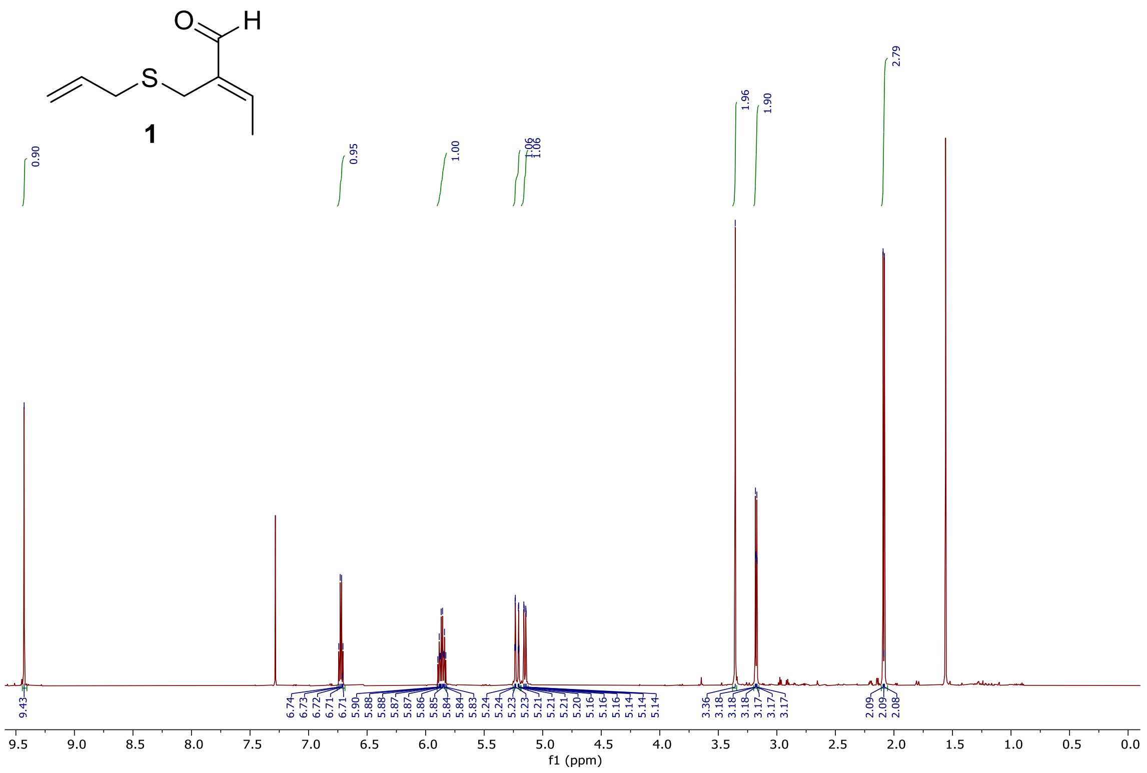
O
H
S
1
0.90
0.95
1.00
1.06
1.06
1.96
1.90
2.79
9.43
6.74
6.73
6.72
6.71
6.71
5.90
5.88
5.88
5.87
5.87
5.86
5.85
5.84
5.84
5.83
5.24
5.23
5.23
5.21
5.21
5.21
5.20
5.16
5.16
5.16
5.14
5.14
5.14
3.36
3.18
3.18
3.18
3.17
3.17
3.17
2.09
2.09
2.08
f1 (ppm)

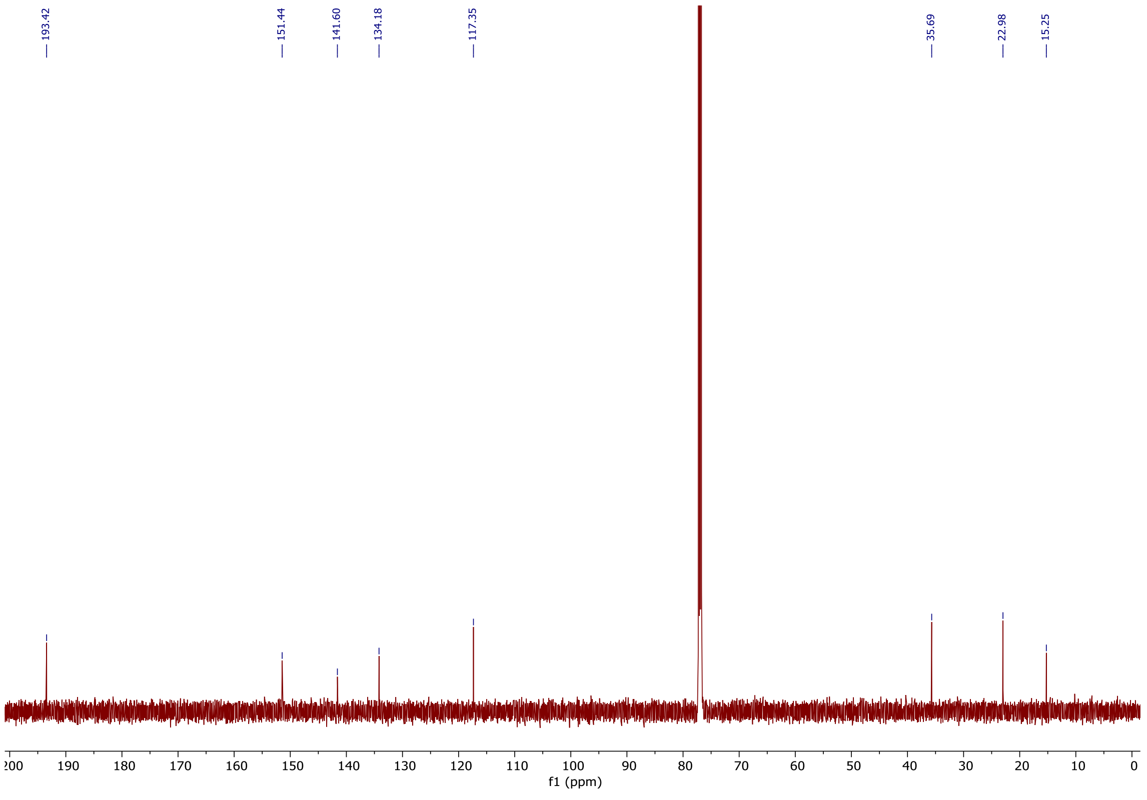
193.42
151.44
141.60
134.18
117.35
35.69
22.98
15.25
f1 (ppm)

1D NOE

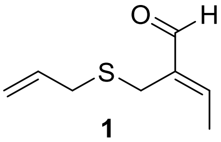
O
H
S

1

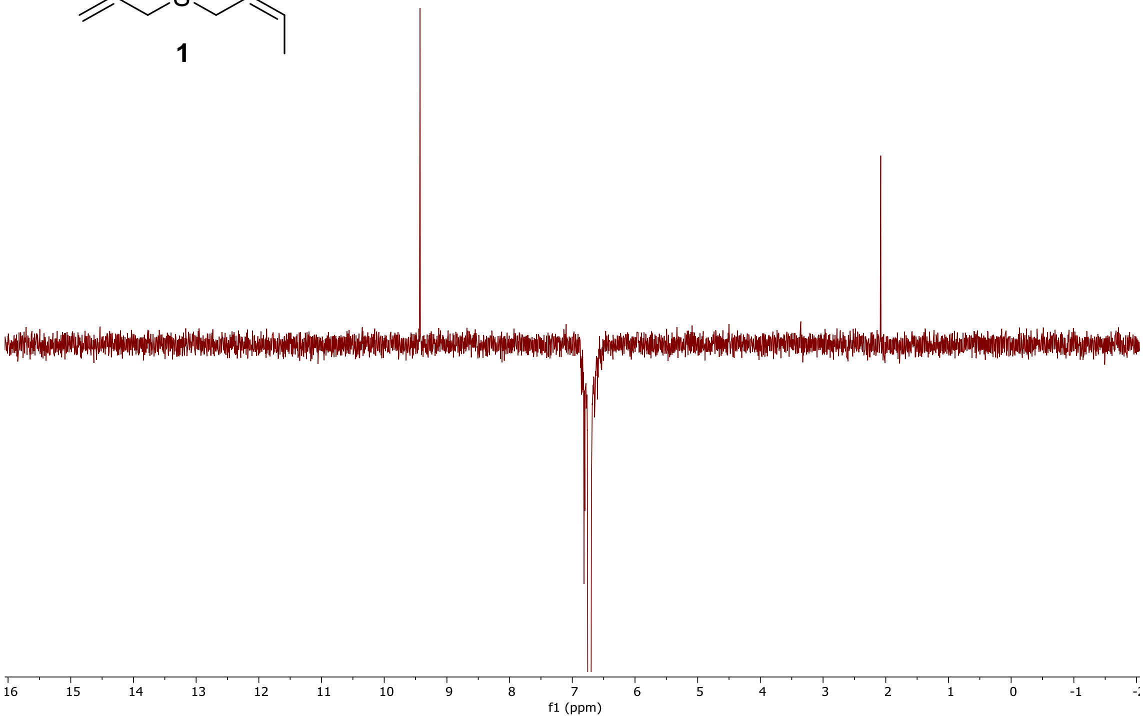
16 15 14 13 12 11 10 9 8 7 6 5 4 3 2 1 0 -1
f1 (ppm)

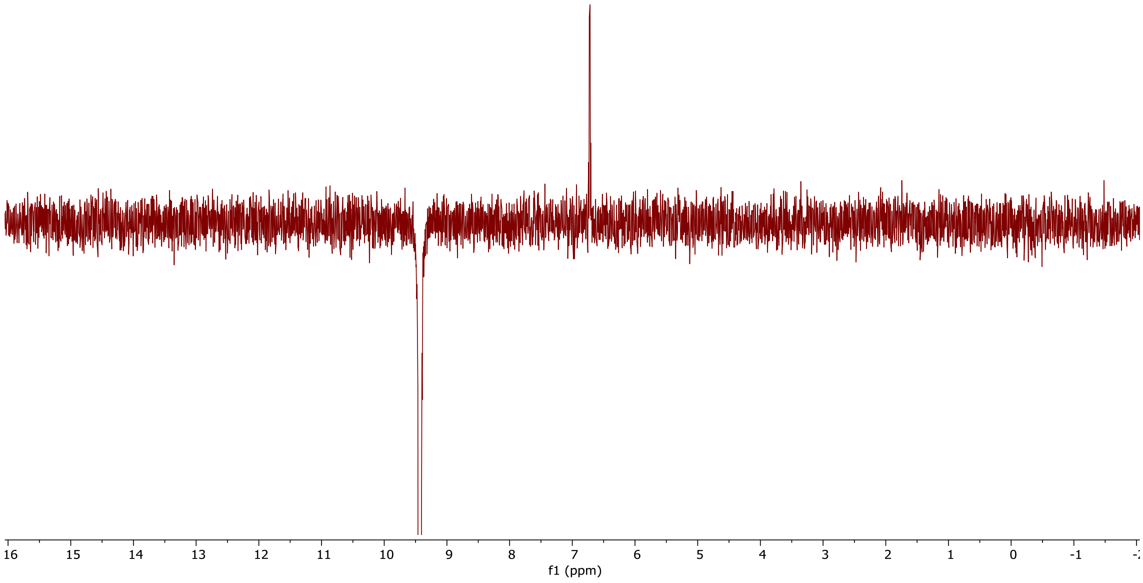
16 15 14 13 12 11 10 9 8 7 6 5 4 3 2 1 0 -1
f1 (ppm)

1D NOE

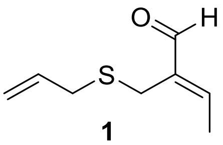

1

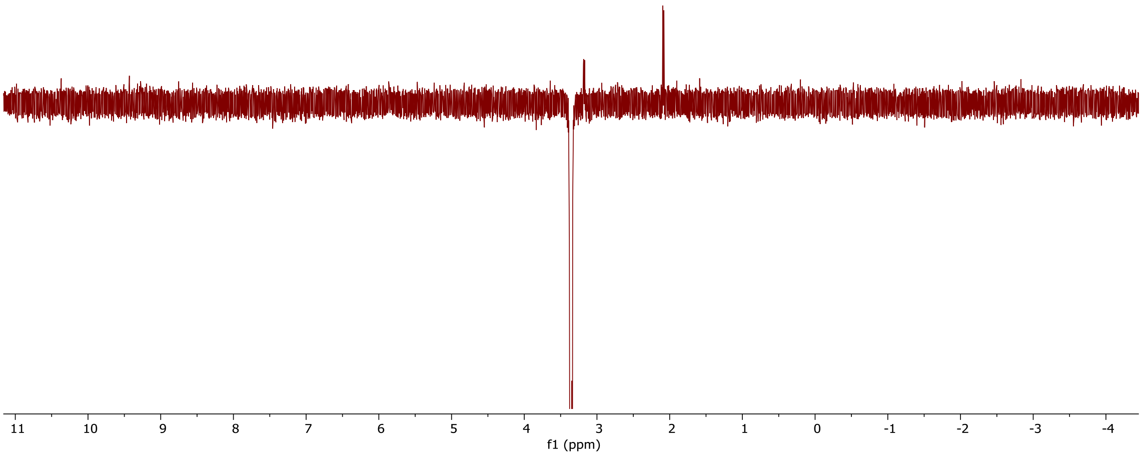

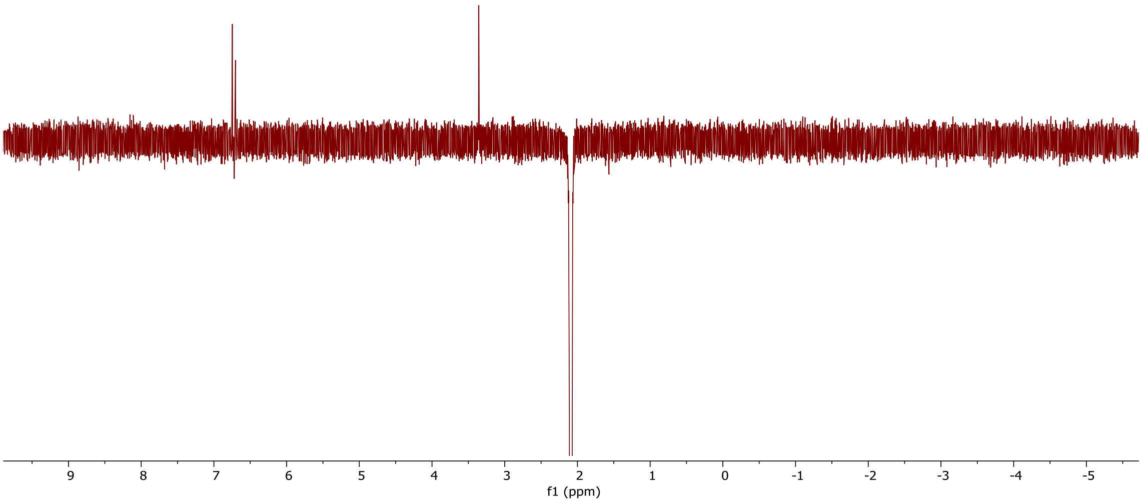

HMBC

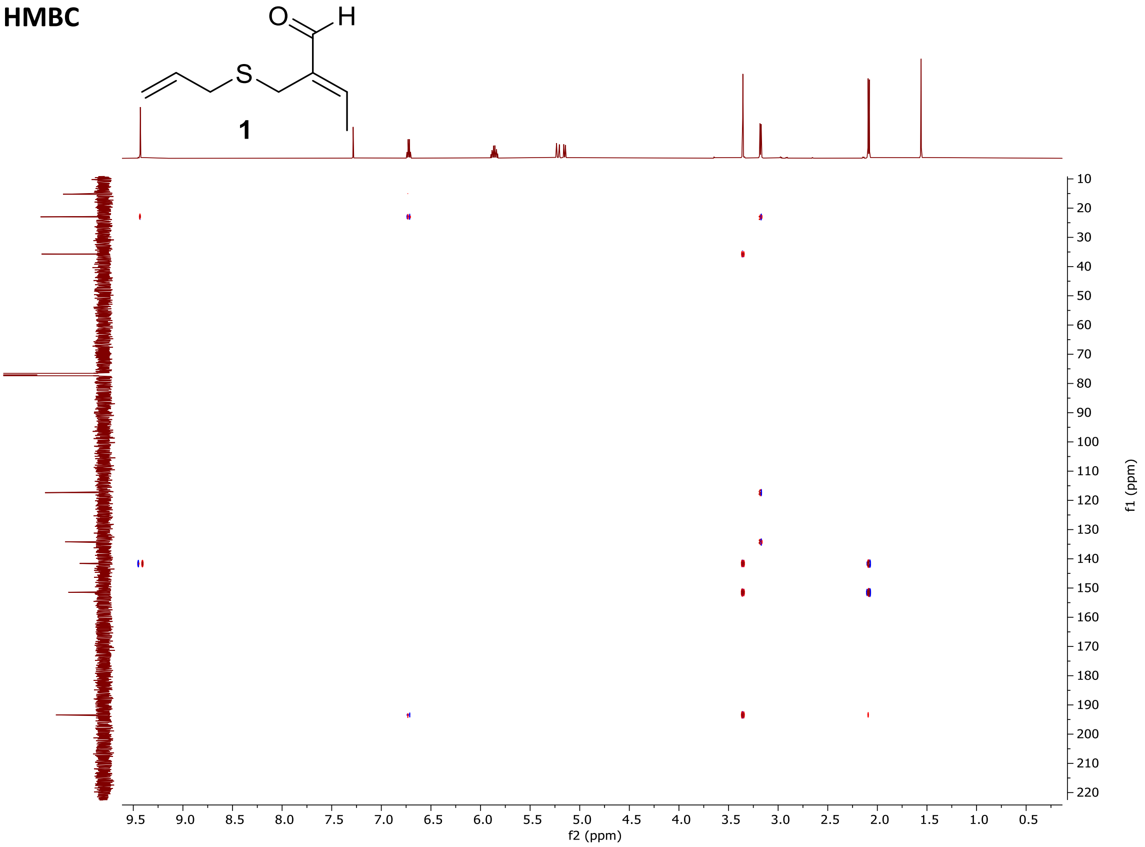
O
H
S
1
9.5 9.0 8.5 8.0 7.5 7.0 6.5 6.0 5.5 5.0 4.5 4.0 3.5 3.0 2.5 2.0 1.5 1.0 0.5
f2 (ppm)
10 20 30 40 50 60 70 80 90 100 110 120 130 140 150 160 170 180 190 200 210 220
f1 (ppm)

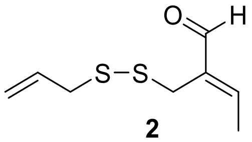
O
H
S
S

2

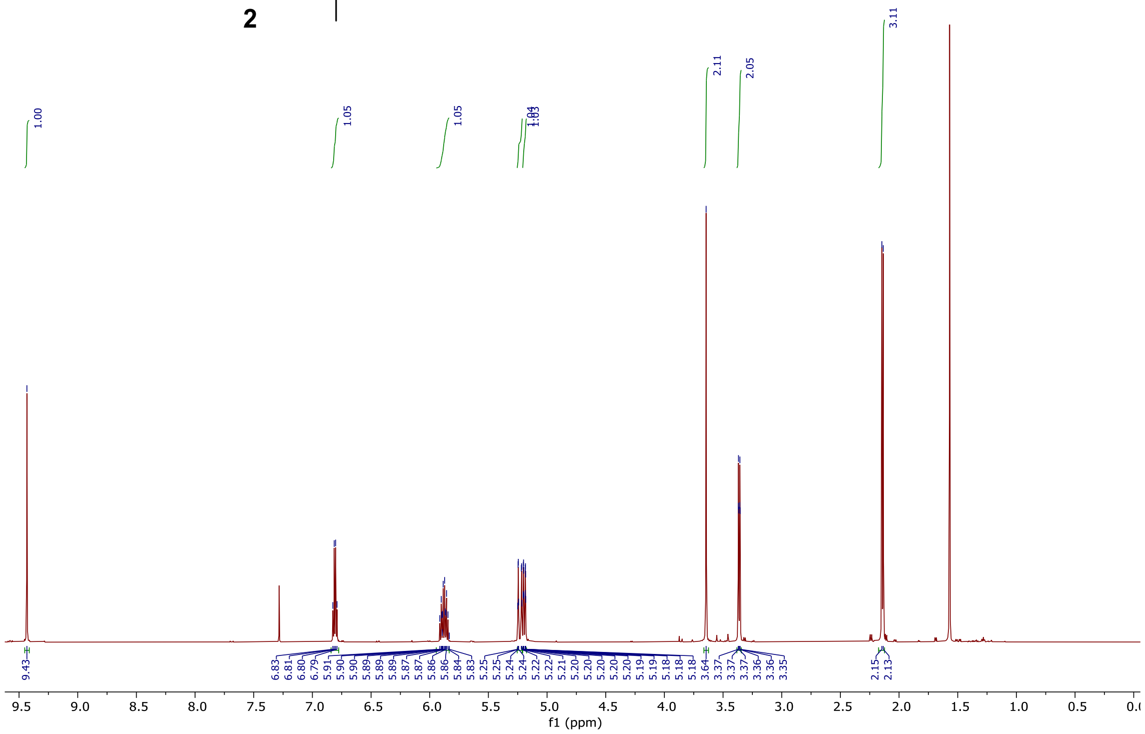
1.00
1.05
1.05
1.04
1.03
2.11
2.05
3.11
9.43
2.15
2.13
9.5
9.0
8.5
8.0
7.5
7.0
6.5
6.0
5.5
5.0
4.5
4.0
3.5
3.0
2.5
2.0
1.5
1.0
0.5
f1 (ppm)

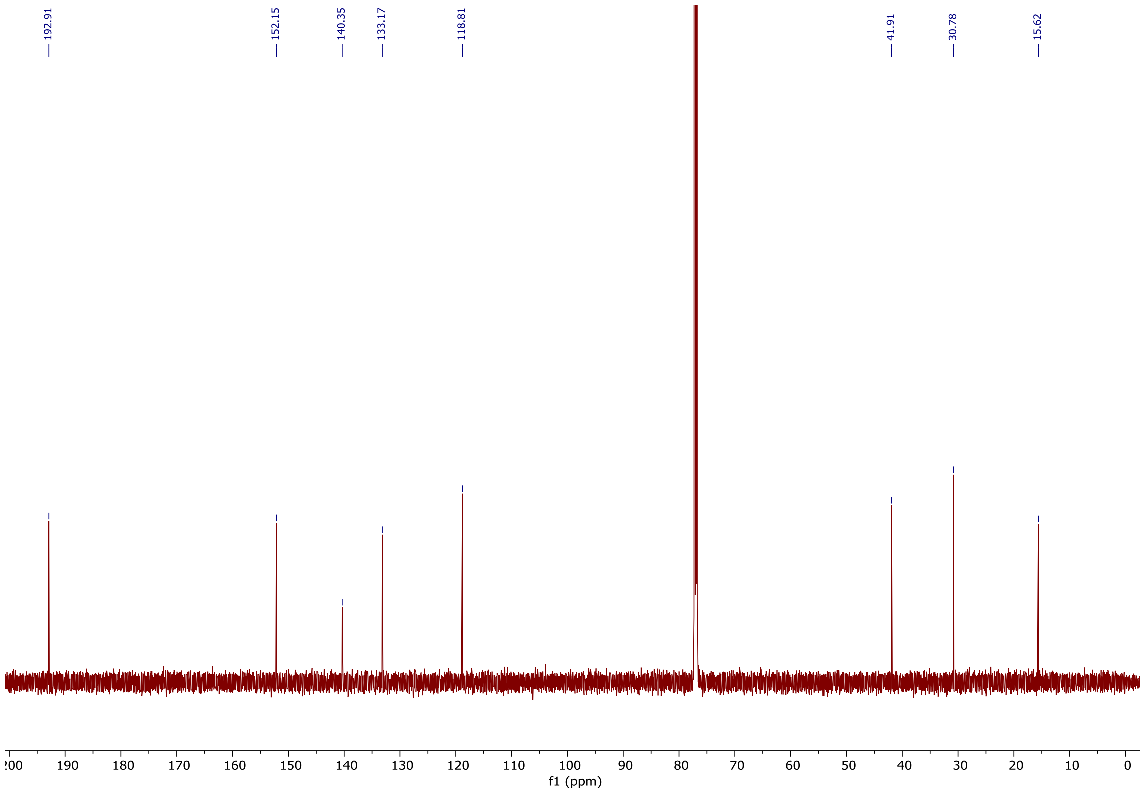
192.91
152.15
140.35
133.17
118.81
41.91
30.78
15.62
190
180
170
160
150
140
130
120
110
100
90
80
70
60
50
40
30
20
10
0
f1 (ppm)

1D NOE

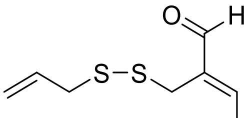
O
H
S
S

2

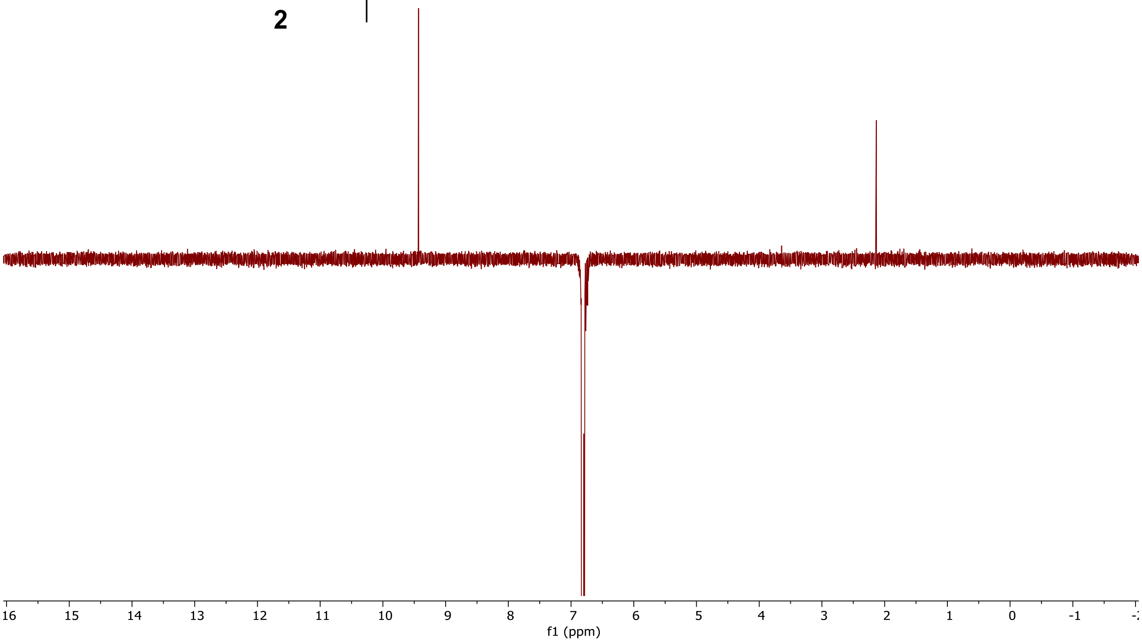
16 15 14 13 12 11 10 9 8 7 6 5 4 3 2 1 0 -1
f1 (ppm)

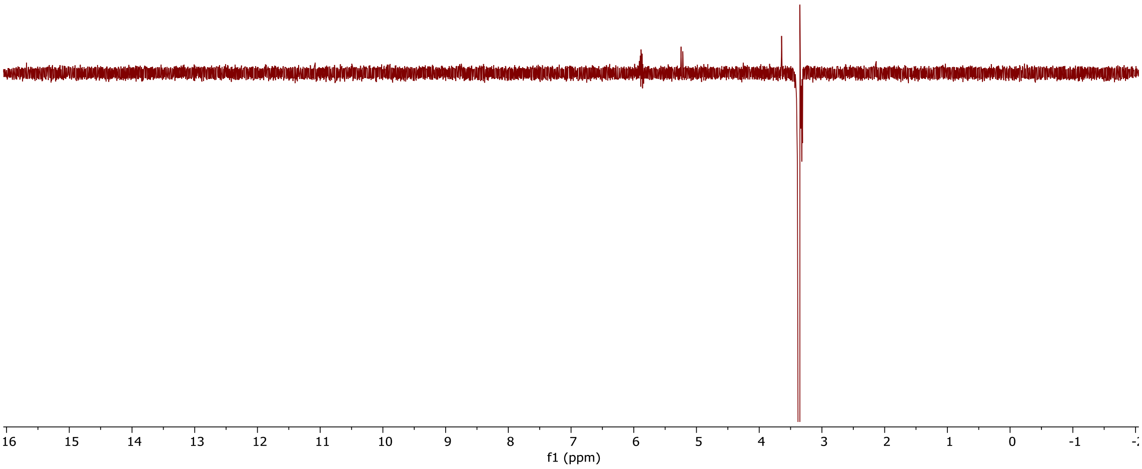
16 15 14 13 12 11 10 9 8 7 6 5 4 3 2 1 0 -1
f1 (ppm)

1D NOE

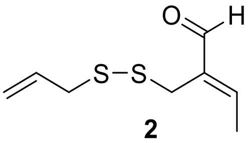
O
H
S
S

2

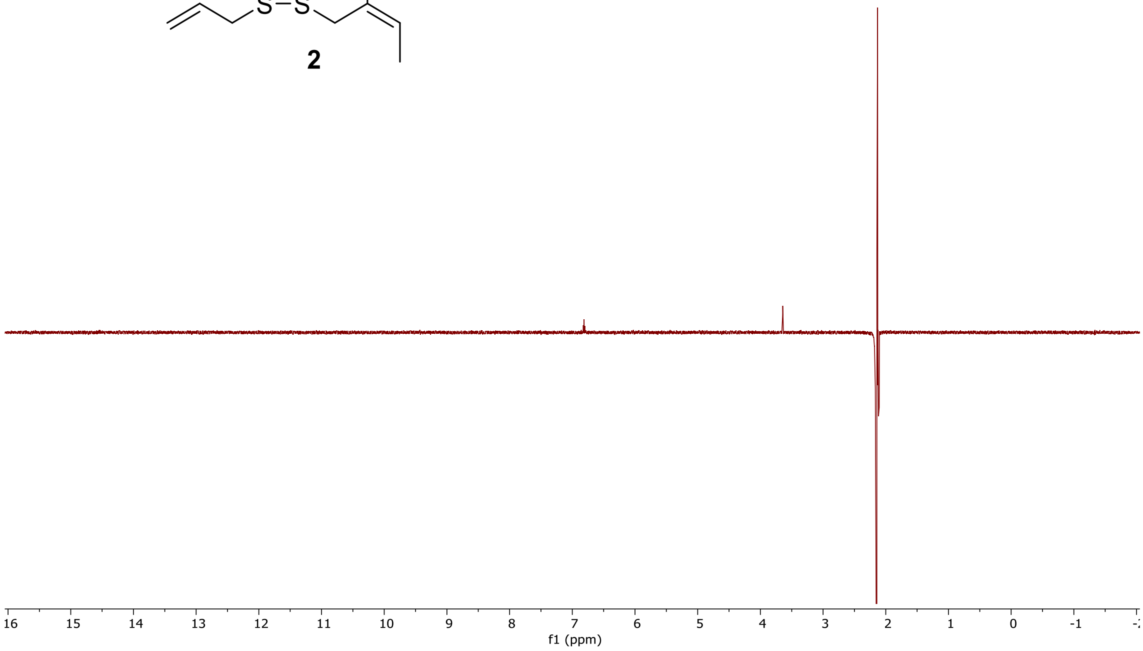
16 15 14 13 12 11 10 9 8 7 6 5 4 3 2 1 0 -1
f1 (ppm)

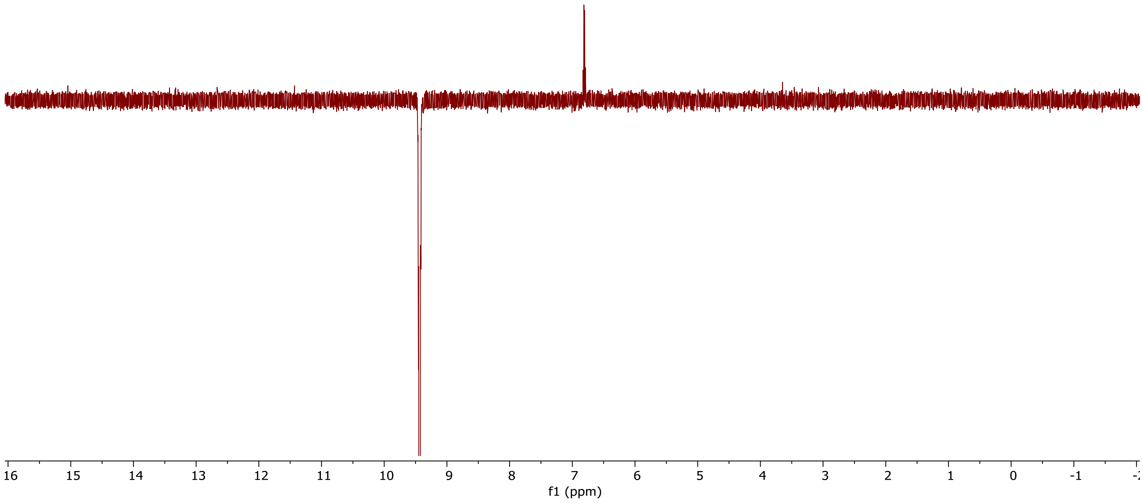
16 15 14 13 12 11 10 9 8 7 6 5 4 3 2 1 0 -1
f1 (ppm)

HMBC

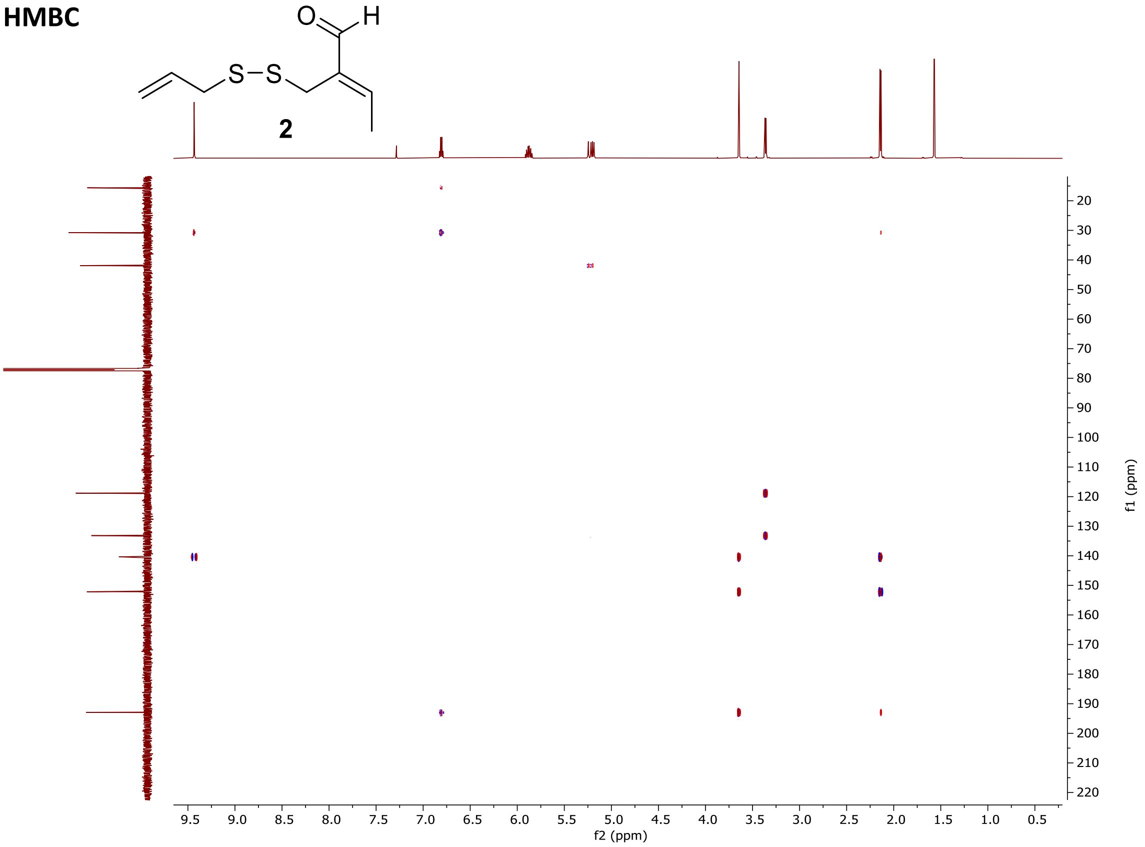

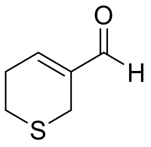

O
H
S

3

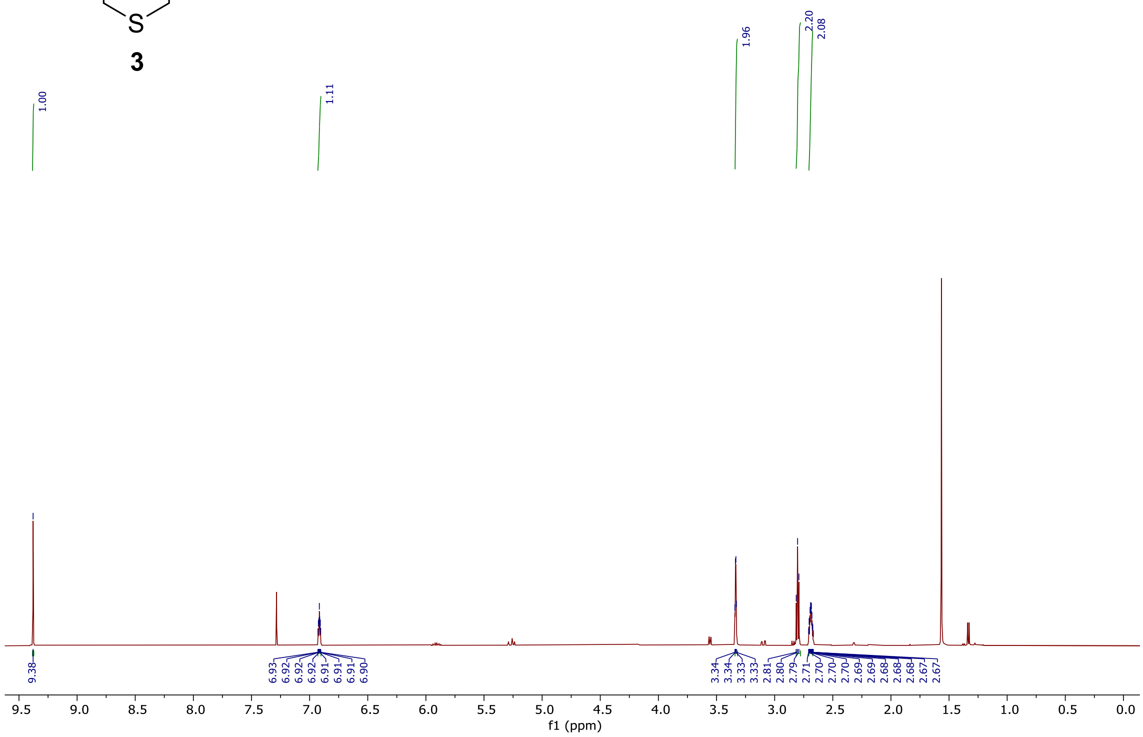

1.00
1.11
1.96
2.20
2.08
9.38
6.93 6.92 6.92 6.92 6.91 6.91 6.91 6.90
3.34 3.34 3.33 3.33
2.81 2.80 2.79
2.71 2.70 2.70 2.70 2.69 2.69 2.68 2.68 2.68 2.67 2.67
9.5 9.0 8.5 8.0 7.5 7.0 6.5 6.0 5.5 5.0 4.5 4.0 3.5 3.0 2.5 2.0 1.5 1.0 0.5 0.0
f1 (ppm)

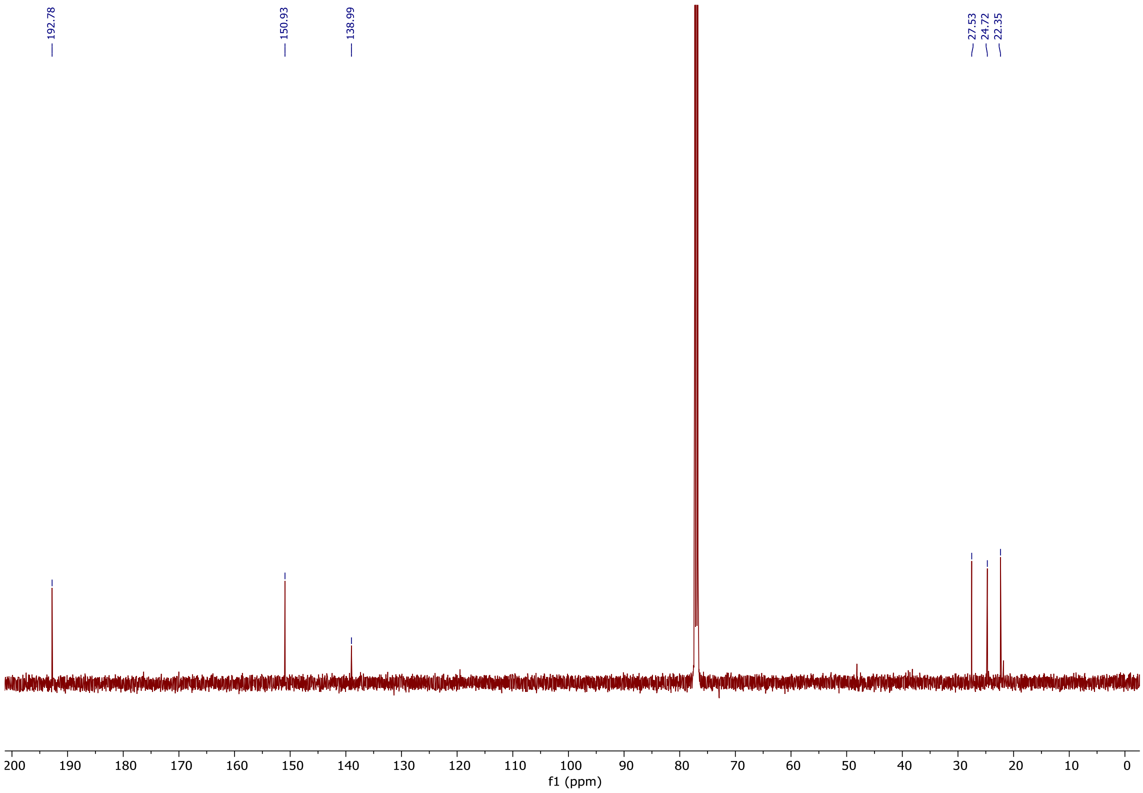

192.78
150.93
138.99
27.53
24.72
22.35
200 190 180 170 160 150 140 130 120 110 100 90 80 70 60 50 40 30 20 10 0
f1 (ppm)

HMBC

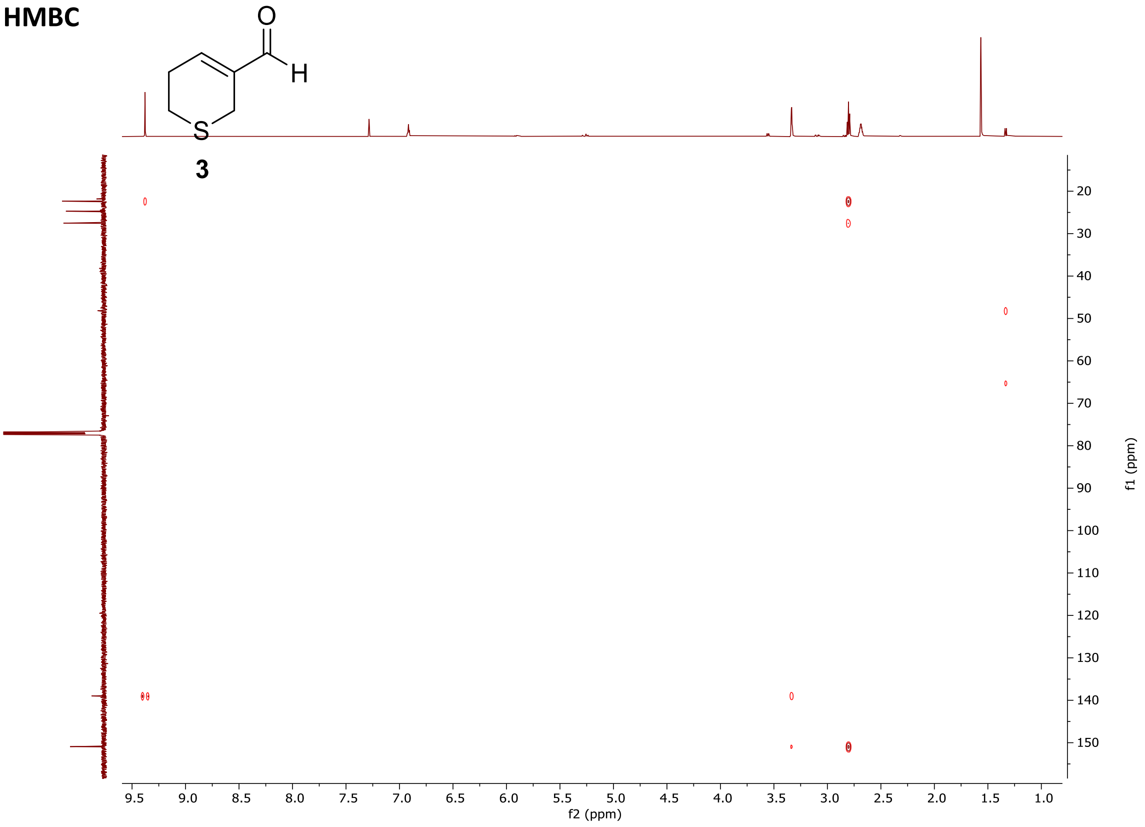

COSY

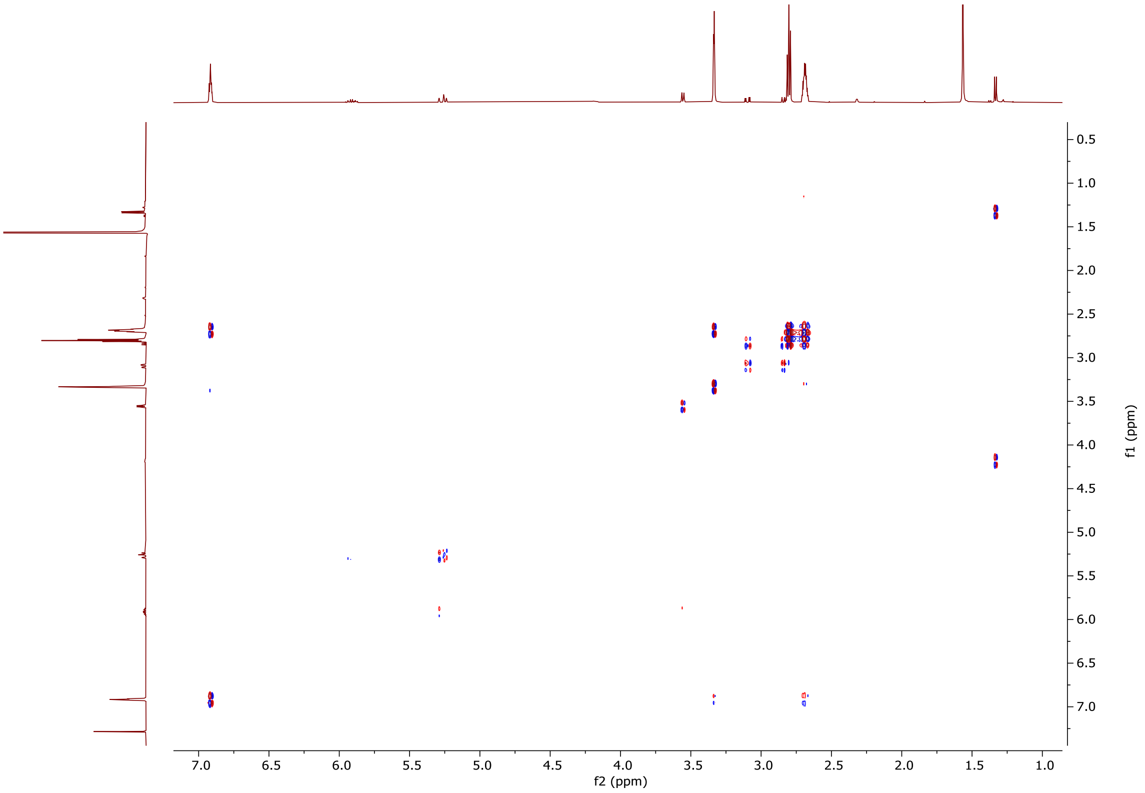

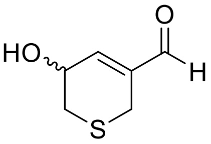
HO
O
H
S

4

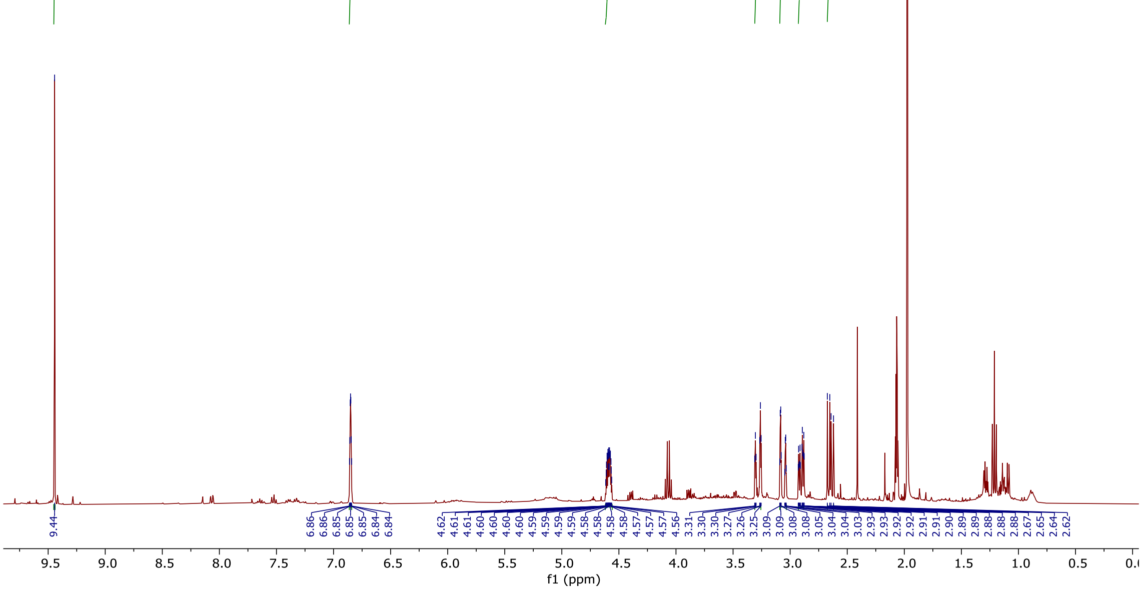
0.94
1.00
1.21
1.12
1.19
1.13
1.11
9.44
f1 (ppm)

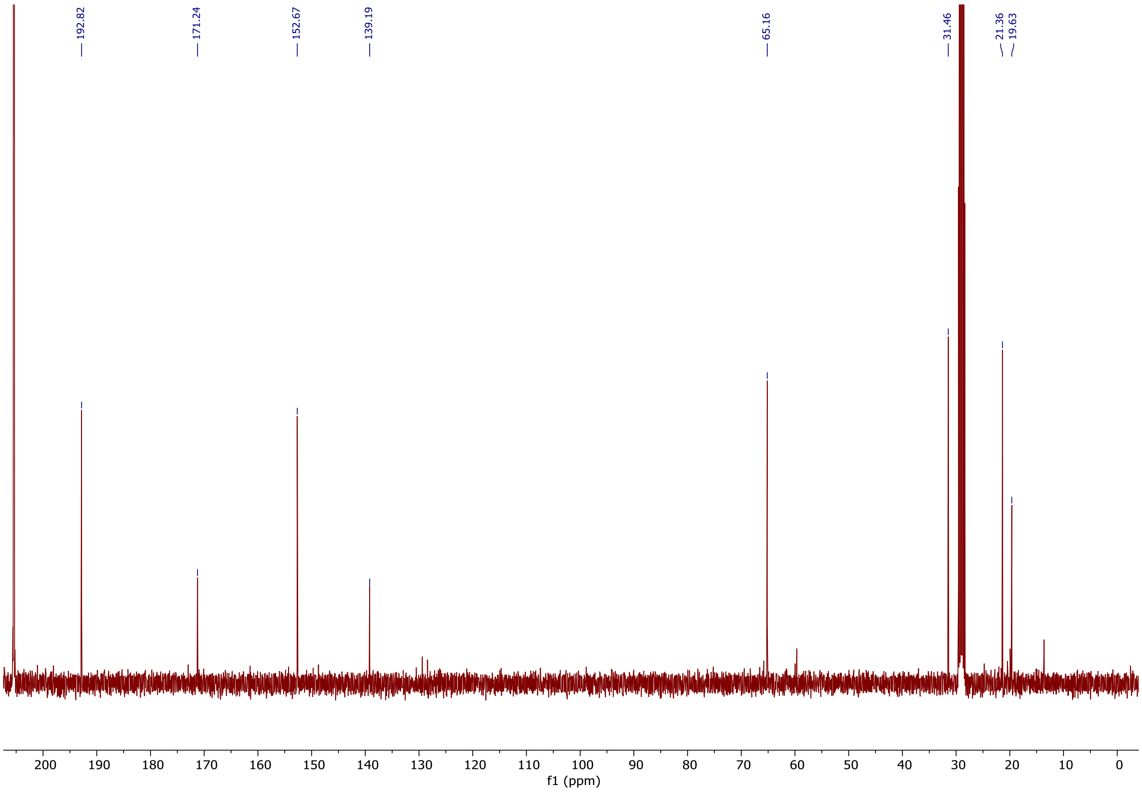
192.82
171.24
152.67
139.19
65.16
31.46
21.36
19.63
f1 (ppm)

HMCB

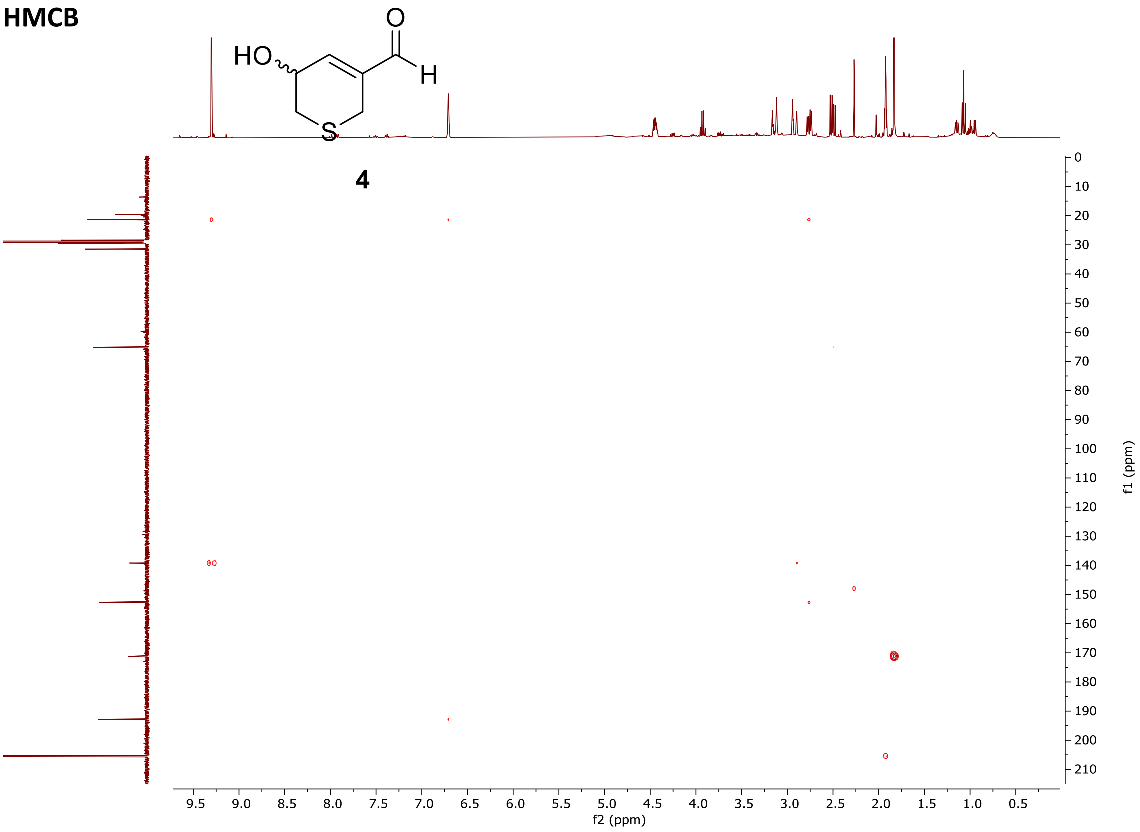

COSY

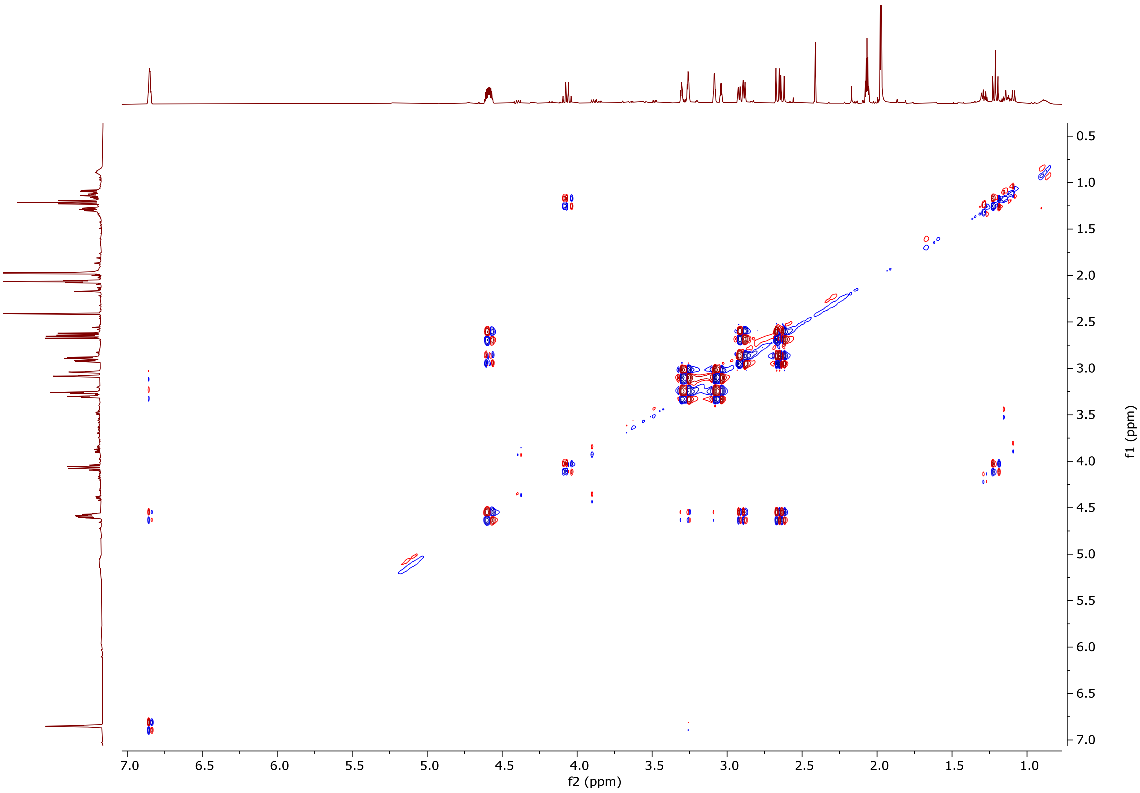

- GC-MS Fraction **A**:

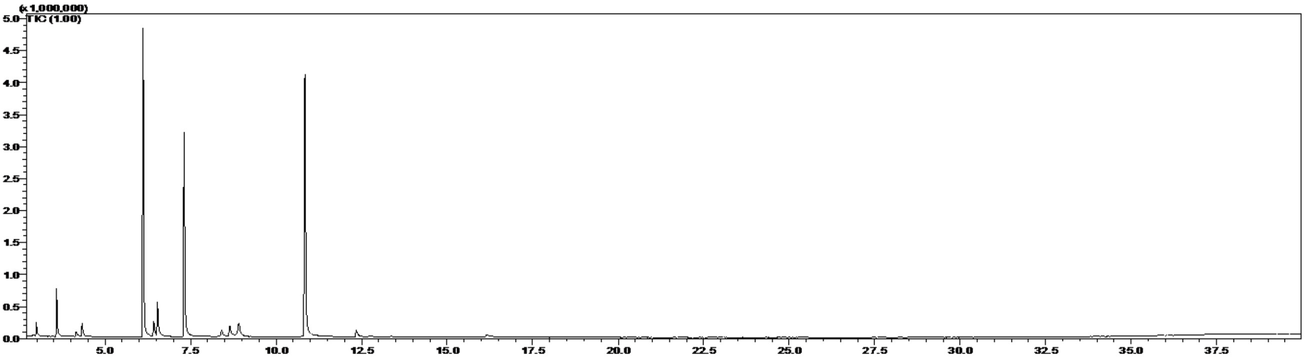

Rt	% Area	Compund
2,90	0.13	1,2-Dithiolane
2,99	1.20	Diallyl sulfide
3,58	3.87	Methyl allyl disulfide
4,15	0.69	3H-1,2-Dithiole
4,33	1.26	Dimethyl trisulfide
4,55	0.08	3-methyl-1-thioetoxy-1,3-butadiene
6,11	28.59	Diallyl disulfide (DADS)
6,42	1.61	Diallyl disulphide Isomer A
6,53	3.76	Diallyl disulphide Isomer B
7,31	21.27	Methyl allyl trisulfide (MATS)
8,41	0.90	3-Vinyl-1,2-dithiacyclohex-4-ene
8,64	1.74	4H-1,2,3-Trithiine
8,91	2.53	2-Vinyl-4H-1,3-dithiine
10,76	0.12	Methane, (methylsulfinyl)(methylthio)-
10,84	30.60	Diallyl trisulfide (DATS)
11,18	0.15	1-Allyl-3-propyltrisulfane
12,34	0.96	5-Methyl-1,2,3,4-tetrathiane
12,72	0.17	Methyl allyl disulfide
16,15	0.39	Diallyl tetrasulfide
